# Transcriptomic and Metabolomic Analysis Reveals That Polystyrene Microplastics Exacerbate Cadmium-Induced Liver Damage in Mice Associated with AMPK-FOXO-Mediated Energy Metabolism Dysregulation

**DOI:** 10.3390/vetsci13080745

**Published:** 2026-07-28

**Authors:** Tong Guo, Yuxue Yang, Fuhao Chen, Haoran Deng, Hongchuan Deng, Xiaoyi Li, Zhuohang Wu, Aoxuan Jiang, Haocheng Huang, Guangneng Peng, Zhijun Zhong, Ziyao Zhou, Kun Zhang, Dechun Chen, Haifeng Liu

**Affiliations:** 1 College of Veterinary Medicine, Sichuan Agricultural University, Chengdu 611130, China; 2 Chongqing Fengdu Agricultural Science and Technology Development Group Co., Ltd., Chongqing 408200, China; 3 People’s Government of Washi Town, Yantan District, Zigong City 643000, China; 4 College of Animal and Veterinary Sciences, Southwest Minzu University, Chengdu 610041, China

**Keywords:** microplastics, cadmium, liver damage, transcriptomics, metabolomics, AMPK-FOXO signaling pathway, energy metabolism

## Abstract

Microplastics (MPs) can adsorb and transport heavy metals, but their influence on cadmium (Cd)-induced hepatotoxicity in mammals remains unclear. Forty-eight male Kunming mice were assigned to control, Cd, MP, and Cd + MP groups and exposed by oral gavage for 42 days. Growth performance, liver injury, oxidative stress, and inflammation were assessed, and transcriptomic and metabolomic analyses were integrated with qPCR, mitochondrial DNA (mtDNA) copy number, and ATP measurements. Compared with Cd alone, combined exposure resulted in greater reductions in body weight gain, the liver index, and antioxidant enzyme activities, together with more severe hepatic lesions and higher levels of liver injury markers and inflammatory cytokines. Co-exposure also induced broader transcriptional and metabolic disturbances than Cd alone. Integrated omics analyses converged on the dysregulation of AMPK–FOXO signaling and related energy metabolic processes. qPCR confirmed more pronounced alterations in pathway-related genes after co-exposure, while reductions in mtDNA copy number and ATP content indicated aggravated mitochondrial dysfunction and impaired energy metabolism. Collectively, these results demonstrate that MPs exacerbate Cd-induced liver injury and suggest that disruption of AMPK–FOXO-associated energy metabolism is a key molecular feature underlying the enhanced hepatotoxicity observed under combined exposure.

## Simple Summary

Microplastics and the toxic metal cadmium are widespread pollutants that often contaminate the same soil, water, and food, so people and animals are frequently exposed to both at once. Because tiny plastic particles can bind and carry heavy metals, the two pollutants together may harm the body more than either does alone. The liver, which breaks down and removes harmful substances, is especially at risk. Most earlier research focused on fish and other aquatic animals, leaving the effects on mammals poorly understood. In this study, we gave mice microplastics, cadmium, or both for six weeks and then examined their livers. The mice exposed to both pollutants had the most severe liver damage, the strongest signs of stress and inflammation, and the largest disturbances in gene and metabolism function. Our results suggest that the combination may disrupt hepatic energy metabolism and impair mitochondrial function through a key regulatory pathway. These findings suggest that microplastics may potentiate cadmium-induced hepatotoxicity in mammals, providing preliminary insight into the potential health risks posed by mixtures of environmental pollutants.

## 1. Introduction

The ongoing advancements in industrialization and urbanization have led to a significant increase in the prevalence of heavy metal pollution, which has become a major environmental concern on a global scale [1]. As a typical environmental pollutant, cadmium (Cd) is characterized by its widespread sources, chemical stability, high mobility in the environment, and long biological half-life. This contaminant has the potential to enter the bodies of humans and animals through various routes. These routes include contact with contaminated soil, water, or food. As a result, this contaminant poses an ongoing threat to public health [2,3,4,5]. In addition, Cd has been identified as a significant heavy metal pollutant in the mining regions of southwestern China. Notably, Sichuan has been documented as having the highest weighted effect value for Cd in this region (ESI = 4.16) [6]. According to the extant research, once Cd enters the body, it has been shown to bind readily to metallothioneins and accumulate in organs such as the liver and kidneys. This process has been shown to induce oxidative stress, mitochondrial dysfunction, inflammatory responses, and cell death, thereby contributing to multi-organ failure [7,8]. Among these, the liver, as a major site for metabolism and detoxification, is one of the key target organs for Cd toxicity [9].

In recent years, microplastics (MPs) have garnered widespread attention as emerging environmental pollutants [10]. MPs typically refer to plastic particles measuring less than 5 mm in diameter. These particles can be produced in two distinct ways: first, they can originate from the degradation of large plastic products; and second, they can be directly generated during industrial production [11,12]. The ubiquity of MPs in water bodies, soil, and the food chain is attributable to their diminutive size, pervasive distribution, and resistance to degradation. These particles have the capacity to enter animal bodies through ingestion and other routes [13]. A mounting body of research suggests that MPs do not merely accumulate within the body but also cause deleterious effects on vital organs, such as the heart, kidneys, and liver, by inducing oxidative stress, inflammatory responses, and organelle damage [14].

It is becoming increasingly evident that MPs should not be regarded as a singular pollutant. Due to their substantial specific surface area and robust adsorption capacity, MPs have the capacity to carry various harmful substances, including heavy metals and pesticides. These substances can potentially alter the migration and transformation processes of these pollutants in the environment, as well as their toxicokinetic behavior within living organisms [15,16]. Consequently, combined exposure to MPs and Cd may produce complex toxicological effects distinct from those of single-substance exposure, potentially even exhibiting synergistic enhancement. Despite the existence of studies on combined MP and Cd exposure, the majority of these studies have focused on aquatic animals and invertebrates. The toxicological endpoints of these studies have been largely centered on oxidative stress, immune damage, and histopathological changes [17,18,19]. However, systematic studies on the mechanisms underlying the development of liver damage in mammals remain limited.

The liver is not only a major target organ for Cd toxicity but also one of the primary sites of potential damage following MP exposure [20]. According to the findings of previous studies, exposure to MPs alone has been demonstrated to cause pathological liver damage. Furthermore, co-exposure to Cd has been shown to exacerbate liver damage [21,22,23]. However, the mechanisms by which MPs influence Cd-induced liver damage in mammals, particularly the metabolic disturbances and molecular regulatory mechanisms involved, have not yet been fully elucidated.

Consequently, this study established a liver injury model by administering polystyrene microplastics (PS-MPs) and CdCl_2_ to mice via oral gavage, either alone or in combination, to characterize the toxicity of combined exposure to MPs and Cd on the livers of mice and their underlying mechanisms. To this end, metabolomics and transcriptomics analyses were integrated. The findings may contribute to a deeper understanding of the biological effects of combined MPs and Cd pollution and offer a theoretical basis for further research on relevant toxicological mechanisms and the development of prevention and control strategies.

## 2. Materials and Methods

### 2.1. Experimental Material

The polystyrene microplastics (PS-MPs) utilized in this study exhibited a diameter of 0.5 μm and were procured from Shanghai Yiyuan Biotechnology Co., Ltd. (Shanghai, China); analytical-grade cadmium chloride (CdCl_2_) was procured from Sinopharm Chemical Reagent Co., Ltd. (Shanghai, China). Both PS-MPs and CdCl_2_ were prepared as working solutions of appropriate concentrations in distilled water for use. According to the manufacturer’s specifications, the PS-MPs have not undergone surface functionalization or chemical modification. Prior to each oral gavage administration, the PS-MP stock solution underwent a 1 min vortex mixing process, followed by 10 min of sonication in an ultrasonic water bath (40 kHz, 25 °C). This procedure was implemented to ensure uniform particle dispersion. The utilization of surfactants and dispersants was eschewed in order to circumvent the possibility of interference with intestinal absorption and bioavailability.

### 2.2. Experimental Animals

Forty-eight SPF-grade male Kunming mice (8 weeks old, weighing 37 ± 3 g) were purchased from Chengdu Dashuo Technology Co., Ltd. (Chengdu, China). All experimental animals were housed under standard environmental conditions: room temperature of 25 °C, good ventilation, a 12 h light-dark cycle, and ad libitum access to food and water. This study was approved by the Animal Care and Use Committee of Sichuan Agricultural University, and all animal housing and handling procedures complied with the relevant regulations on laboratory animal management at Sichuan Agricultural University (Approval no. SYXK 2022-166).

After a 1-week acclimatization period, the 48 mice were randomly divided into four groups of 12 mice each: the control group, the Cd group, the MP group, and the Cd + MP group (Table 1). The oral gavage doses of CdCl_2_ and MPs were based on previous studies, at 4.8 mg/kg body weight and 10 mg/kg body weight, respectively. Administration was performed once daily at 12:00 via oral gavage for 42 consecutive days [24,25]. The mental state and growth of the mice were observed daily during the experiment.

Subsequent to the procedure, following a 12 h fast and water deprivation, the body weights of the mice were documented, and blood samples were obtained via the orbital vein. The blood samples were then permitted to stand at room temperature for a period of two hours. Subsequently, the blood was subjected to a centrifugal process at 4000× *g* for a duration of 15 min, with the objective of effectuating the separation of the serum. The serum was subsequently stored at a temperature of –80 °C, pending subsequent utilization. Subsequently, the livers were dissected and weighed. Following a rinse with phosphate-buffered saline (PBS) or 0.9% saline, the samples were rapidly frozen in liquid nitrogen for a duration of 30 min. Thereafter, they were stored at −80 °C for subsequent analysis. The calculation of body weight gain and liver index was performed using the following formulas: [(final weight − initial weight)/initial weight] × 100%; (liver weight/body weight) × 100%.

### 2.3. Scanning Electron Microscopy of PS-MPs

In order to evaluate the morphology and surface characteristics of PS-MPs, 1 mL of the PS-MPs working solution was transferred into a centrifuge tube and centrifuged at 10,000 rpm for 20 min using a centrifuge pre-cooled to 4 °C. After centrifugation, the supernatant was discarded, and the precipitate was collected. The precipitate was then dried in a constant-temperature oven at 60–70 °C to obtain PS-MP powder samples, which were subsequently examined using a scanning electron microscope.

### 2.4. Hematoxylin–Eosin Staining

Liver tissue was fixed in 4% paraformaldehyde for 24 h, routinely embedded in paraffin, and sectioned into 5 μm thick sections. The sections were stained with hematoxylin and eosin (H&E) and examined histopathologically. Using Image J software (version: 2.11.0), five fields of view were randomly selected from each group of sections to count the number of inflammatory cells. The proportion of inflammatory cells was calculated relative to the total number of cell nuclei within the same field of view to assess the degree of inflammatory cell infiltration and tissue damage.

### 2.5. Biochemical Analysis

Liver tissue was homogenized with PBS at a 1:9 (*w*/*v*) ratio and centrifuged at 2500× *g* for 10 min, and the supernatant was collected. Alanine aminotransferase (ALT) and aspartate aminotransferase (AST) activities in the liver tissue were measured according to the instructions of the respective kits (Geruisi Bio Co., Ltd., Suzhou, China). For oxidative stress-related parameters in serum, superoxide dismutase (SOD) and catalase (CAT) activities were measured using a commercially available kit (Jiancheng Biotech Co., Ltd., Nanjing, China); serum levels of interleukin-6 (IL-6) and tumor necrosis factor-α (TNF-α) were measured using an ELISA kit (Jiancheng Biotech Co., Ltd., Nanjing, China).

### 2.6. Transcriptomic Analysis

#### 2.6.1. RNA Extraction and Quality Assessment

Total RNA was extracted from approximately 50 mg of frozen liver tissue using TRIzol reagent (Qiagen, Hilden, Germany) according to the manufacturer’s instructions (*n* = 3). The sample size for each group was three, with samples selected using the random number sequence method. RNA concentration and purity were measured using a NanoDrop ND-2000 spectrophotometer (Thermo Fisher Scientific, Waltham, MA, USA), and RNA integrity was assessed using an Agilent 4150 Bioanalyzer (Agilent Technologies, Santa Clara, CA, USA). Only samples with an OD260/280 ratio of 1.8–2.2 and an RQN > 6.5 were selected for subsequent sequencing.

#### 2.6.2. Library Construction and RNA Sequencing

Transcriptome libraries were constructed using the Illumina Stranded mRNA Prep Kit (Illumina, San Diego, CA, USA). Poly(A)-tailed mRNA was enriched using magnetic beads conjugated with oligo(dT) and randomly fragmented into approximately 300 bp fragments. Subsequently, first- and second-strand cDNA synthesis, terminal repair, 3′ A-tail addition, adapter ligation, and PCR amplification were performed sequentially, followed by purification using AMPure XP magnetic beads (Beckman Coulter, Brea, CA, USA). Library quality and concentration were assessed using a Qubit 4.0 fluorometer and an Agilent Bioanalyzer 4150, respectively. Qualified libraries were sequenced using 150 bp paired-end sequencing on the Illumina NovaSeq X Plus platform (Illumina, USA).

#### 2.6.3. Quality Control and Sequence Alignment

Raw sequencing data underwent quality control using FastQC (v0.12.1) and Trimmomatic (v0.39), with adapter sequences, low-quality bases, and reads containing >5% ambiguous nucleotides removed. The filtered clean reads were aligned to the mouse reference genome (GRCm39, Ensembl) using HISAT2 (v2.2.1), and gene expression levels were quantified using RSEM (v1.3.3), with results expressed as TPM.

#### 2.6.4. Differential Expression Analysis

DESeq2 (v1.36.0) in R was used to identify differentially expressed genes (DEGs), with *p* < 0.05 and |log2Fold Change| ≥ 1 as the criteria for significant differential expression. Three group comparisons were performed: Cd group vs. Con group, Cd + MP group vs. Con group, and Cd + MP group vs. Cd group, to assess the effect of MPs on the gene expression profile associated with Cd-induced liver injury. To evaluate the biological reproducibility among samples, principal component analysis (PCA) and Pearson correlation analysis were performed using R (v4.2.0).

#### 2.6.5. Functional Annotation and Enrichment Analysis

GOATOOLS and KOBAS (v3.0) were used to perform Gene Ontology (GO) and Kyoto Encyclopedia of Genes and Genomes (KEGG) functional annotation of DEGs. The GO analysis covered three levels: biological processes (BP), cellular components (CC), and molecular functions (MF). Enrichment analysis was performed using Fisher’s exact test, with false discovery rate (FDR) correction applied via the Benjamini–Hochberg method; results with an FDR < 0.05 were considered significantly enriched.

### 2.7. Metabolomics Analysis

#### 2.7.1. Metabolite Extraction

The liver tissue was weighed, with an approximate measurement of 50 milligrams being recorded (*n* = 7). The sample size for each group was seven, with samples selected using the random number sequence method. The tissue was then placed in a 2 milliliter centrifuge tube, which contained 1–2 pre-chilled 6 mm stainless steel beads. Next, 400 μL of extraction solution (methanol: water = 4:1, *v*/*v*) containing 0.02 mg/mL of L-2-chlorophenylalanine as an internal standard was added. The sample was processed at −20 °C using a Wonbio-96C tissue homogenizer (Shanghai WB Biotechnology Co., Ltd., Shanghai, China) at 60 Hz for 5 min, followed by ultrasonication in an ice-water bath for 30 min (40 kHz, 5 °C). The sample was allowed to stand at −20 °C for 30 min, followed by centrifugation at 4 °C and 13,000× *g* for 15 min. The supernatant of the sample was collected and transferred to vials for subsequent storage at −80 °C. Liquid chromatography-mass spectrometry (LC-MS) analysis was then performed on the stored sample. Equal volumes (10 μL) of the culture medium from each sample were mixed to prepare quality control (QC) samples. The insertion of one QC sample for every 10 to 15 samples injected during the analysis is recommended. This practice allows for the monitoring of instrument stability. The QC performance criteria are as follows: the relative standard deviation (RSD) of the metabolic markers detected in the QC samples must be less than 30%; the retention time shift in consecutive QC injections must be less than 0.1 min; and the mass accuracy must be less than 5 ppm.

#### 2.7.2. Liquid Chromatography-Mass Spectrometry/Mass Spectrometry

The untargeted metabolomics analysis was performed using an ultra-high-performance liquid chromatography (UHPLC) system coupled with a Q Exactive HF-X mass spectrometer (Thermo Fisher Scientific, USA), with an HSS T3 C18 column (100 mm × 2.1 mm, 1.8 μm; Waters, Waltham, MA, USA). The column temperature was set to 40 °C, the flow rate to 0.4 mL/min, and the injection volume to 3 μL. Mobile phase A was composed of water containing 0.1% formic acid, while mobile phase B consisted of acetonitrile, isopropanol, and water (47.5:47.5:5, by volume) containing 0.1% formic acid. The mass spectrometry detection process involved the concurrent utilization of both positive and negative ion electrospray ionization modes (ESI+ and ESI−, respectively). The scan range extended from *m*/*z* 70 to 1050, with a spray voltage of ±3.5 kV and a capillary temperature maintained at 325 °C.

#### 2.7.3. Data Processing and Metabolite Identification

The raw data were processed using Progenesis QI (Waters, USA) for peak extraction, peak alignment, deconvolution, and normalization. The resulting feature peaks were then compared against HMDB, METLIN, and an in-house database. A mass error of ≤10 ppm was used as the standard for metabolite identification. The metabolite intensities were then normalized against an internal standard and subsequently log-transformed to ensure the integrity of subsequent statistical analysis.

#### 2.7.4. Multivariate Statistical Analysis

Principal component analysis (PCA) and partial least squares discriminant analysis (PLS-DA) were performed using SIMCA-P (v14.1, Umetrics, Umeå, Sweden) to assess sample clustering trends and the separation of groups. The stability of the model was confirmed through 200 bootstrap repetitions. The selection of metabolites that exhibited differential expression was predicated on the criteria of variable importance in projection (VIP) > 1 and *p* < 0.05 (Student’s *t*-test). The data were presented in the form of volcano plots, hierarchical clustering heatmaps, and correlation matrices, which were then visualized using R (v4.2.0). The intergroup comparisons were executed in a manner consistent with the protocol outlined in the transcriptomics analysis section.

#### 2.7.5. Metabolic Pathways and Enrichment Analysis

Metabolites that demonstrated significant differential expression were mapped to the KEGG database for metabolic pathway annotation. The pathway enrichment analysis was performed using MetaboAnalyst 5.0 and the Python SciPy module. Significantly enriched pathways were identified based on Fisher’s exact test combined with FDR correction. An FDR threshold of <0.05 was used as the significance criterion.

### 2.8. Validation of Transcriptomics and Metabolomics Results

Signaling pathways identified through transcriptomics and metabolomics were analyzed, after which key pathways were selected for validation.

#### 2.8.1. qRT-PCR

The RNA extraction method employed for quantitative real-time polymerase chain reaction (qRT-PCR) was consistent with the method utilized for transcriptomic analysis. Subsequent to extraction, the purity of the RNA was assessed using a NanoDrop spectrophotometer. Only samples with an OD260/280 ratio of 1.8–2.2 were selected for subsequent experiments. A quantity of total RNA ranging from 1000 to 5000 nanograms was mixed with 2 microliters of oligo(dT)16 primer. The mixture was subsequently diluted to 14.5 μL with RNase-free deionized water. The mixture was then incubated at 70 °C for 5 min and rapidly cooled on ice for 2 min. Thereafter, 5× M-MLV buffer, deoxynucleoside triphosphates, RNase, and M-MLV reverse transcriptase were added. The mixture was then incubated at 42 °C for 60 min. The reaction was terminated by heating at 95 °C for 5 min. Subsequent to the generation of cDNA, it was utilized in immediate experiments or stored at −80 °C. qPCR primers were designed using NCBI Primer-BLAST based on the mRNA sequences of target genes and synthesized by Shanghai Bioengineering Co., Ltd. (Shanghai, China). The genes detected included molecules associated with the AMPK-FOXO signaling pathway, as identified by integrated transcriptomic and metabolomic analysis. The genes identified as significantly altered included Cpt1a, Cd36, Acacb, Pik3r1, Irs2, Gadd45g, Fbxo32, Bcl6, and Ccng2. The primer sequences are delineated in Table 2.

The total volume of the qPCR reaction per well was 20 μL, consisting of 10 μL of 2× SYBR Green premix, 0.4 μL of forward primer (10 μm), 0.4 μL of reverse primer (10 μm), 1 μL of cDNA template, and RNase-free water to make up the volume. The amplification process was carried out using a real-time quantitative PCR instrument (Kunpeng, Beijing, China), following the designated program: the initial denaturation step was performed at 94 °C for 30 s, followed by 40 cycles of 5 s denaturation at 94 °C and 30 s extension at 60 °C. The protocol was concluded with a melting curve analysis, which involved heating the samples from 60 °C to 95 °C at a rate of 0.5 °C per cycle. This analysis was performed to verify the specificity of the amplification reaction. The relative gene expression levels were calculated using the 2^−ΔΔCt^ method and normalized using GAPDH as the internal control.

#### 2.8.2. Determination of Mitochondrial DNA (mtDNA) Copy Number and ATP Content

The quantity of mitochondrial DNA (mtDNA) in liver tissue was ascertained through the implementation of quantitative PCR (qPCR). The procedure was performed in accordance with the instructions for the Mouse Mitochondrial DNA Dye-Based qPCR Kit (Suzhou Greys Biotechnology Co., Ltd., Suzhou, China), and the experimental workflow was consistent with the qPCR method described above. An ATP assay kit (Bio-Tech Institute, Shanghai, China) was utilized to assess the levels of adenosine triphosphate (ATP) in liver tissue. Approximately 100 mg of liver tissue was homogenized with 500 μL of pre-chilled ATP lysis buffer. The homogenate was then subjected to centrifugation at 12,000× *g* for 10 min at room temperature. The supernatant was subsequently collected, and the pH was adjusted to a range of 6.5–8.0. For the assay, 100 μL of ATP detection reagent was added to each well of a 96-well plate. Following a five-minute equilibration period at room temperature, 20 μL of the sample extract was added, and the resulting luminescence intensity was promptly measured using a microplate reader (Thermo Fisher Scientific, USA). A standard curve was plotted using ATP standards, and ATP levels were normalized based on BCA protein quantification results.

### 2.9. Statistical Analysis

For the data in Section 2.4 and Section 2.5, a two-way analysis of variance (two-way ANOVA) was conducted using Cd exposure (yes/no) and MP exposure (yes/no) as two independent factors. The treatment groups corresponding to each factor were as follows: the Con group (Cd−, MPs−), the MP group (Cd−, MPs+), the Cd group (Cd+, MPs−), and the Cd + MP group (Cd+, MPs+). In Section 2.8, one-way analysis of variance (ANOVA) was employed. All data analysis and graphing were performed using SPSS and GraphPad Prism 9.0 software. The results obtained are expressed as the mean value ± standard deviation (mean ± SD). A two-tailed Student’s *t*-test was employed to compare pairs of groups; if the results were significant, Tukey’s post hoc multiple comparison test was subsequently performed. Statistically speaking, a *p*-value less than 0.05 was deemed to be significant. The following labeling system was employed for the significance levels in the figures: *: *p* < 0.05, **: *p* < 0.01, ***: *p* < 0.001.

## 3. Results

### 3.1. Surface Characteristics of PS-MPs

**Figure 1:**
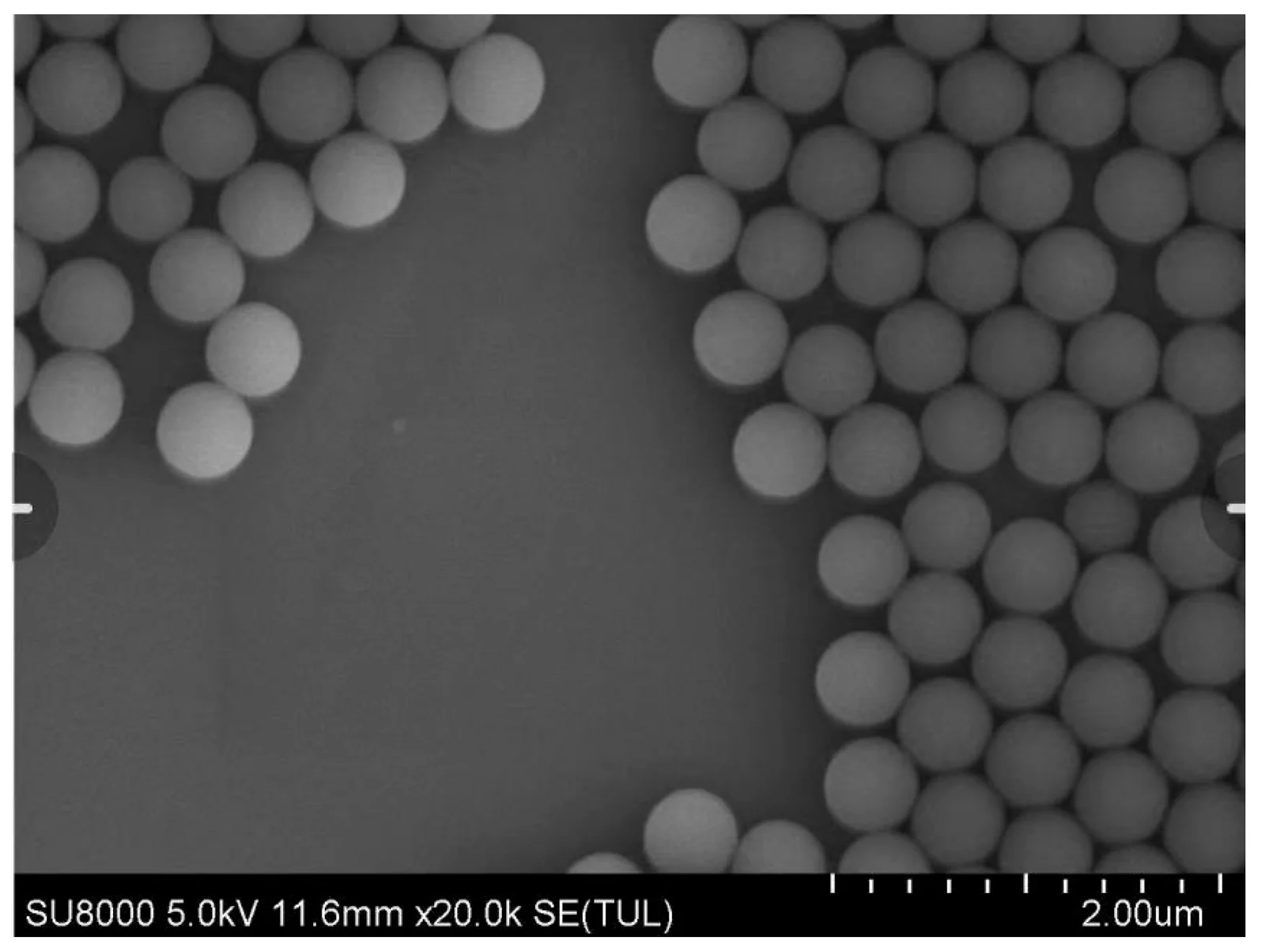
Scanning electron microscope (SEM) image of PS-MPs.

To characterize the surface features and physicochemical properties of PS-MPs, they were examined using a scanning electron microscope. The results demonstrated that the PS-MPs particles exhibited smooth surfaces and were closely packed, uniform in size, and regular in shape, forming spherical particles (Figure 1).

### 3.2. MPs Exacerbate the Negative Effects of Cd on Growth Performance in Mice

**Figure 2:**
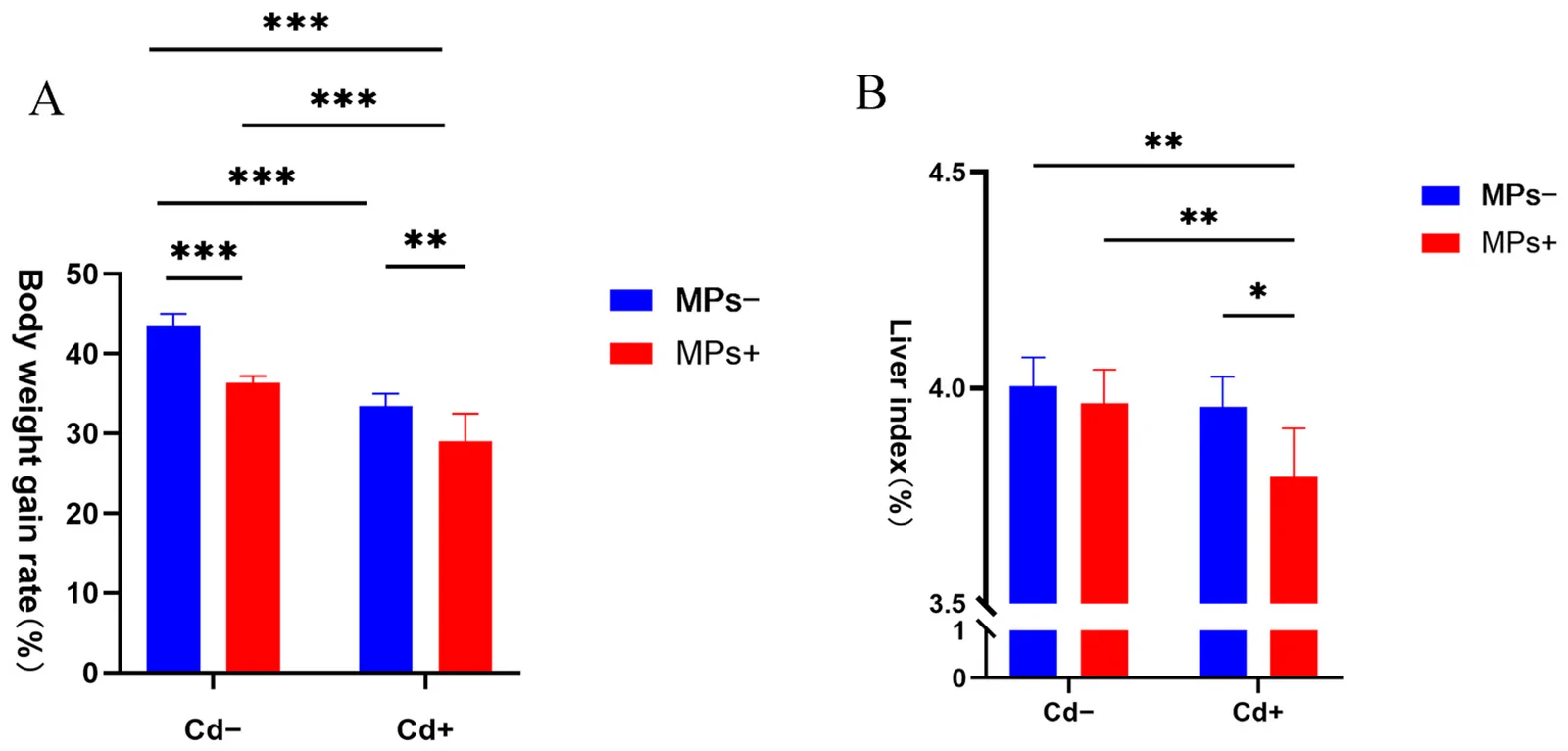
Effects of MPs and Cd on growth performance in mice. (**A**) Body weight gain rate in mice; (**B**) liver index in mice. “*” indicates *p* < 0.05; “**” indicates *p* < 0.01; “***” indicates *p* < 0.001.

In order to evaluate the effects of exposure to MPs and Cd, either alone or in combination, on the growth performance of mice, body weight gain and liver index were measured. In comparison with the Con group, a significant reduction in body weight gain was observed in the Cd, MP, and Cd + MP groups (*p* < 0.05), with the Cd + MP group demonstrating the most substantial decrease. This discrepancy was further accentuated in comparison with the single-exposure groups (Figure 2A, *p* < 0.01). There was no significant difference in body weight gain rates between the two single-exposure groups. The liver index is a metric employed in the evaluation of hepatic damage in murine models. The results demonstrated that, in comparison with the Con group, both the Cd and MP groups exhibited a downward trend, though these differences did not attain statistical significance. However, the liver index in the Cd + MP group was significantly lower than that in the other groups (Figure 2B, *p* < 0.05). The results of the present study indicate that both MPs and Cd had a detrimental effect on the growth performance of mice. This negative effect was more pronounced when MPs and Cd were present simultaneously.

### 3.3. MPs Exacerbate Cd-Induced Liver Damage

**Figure 3:**
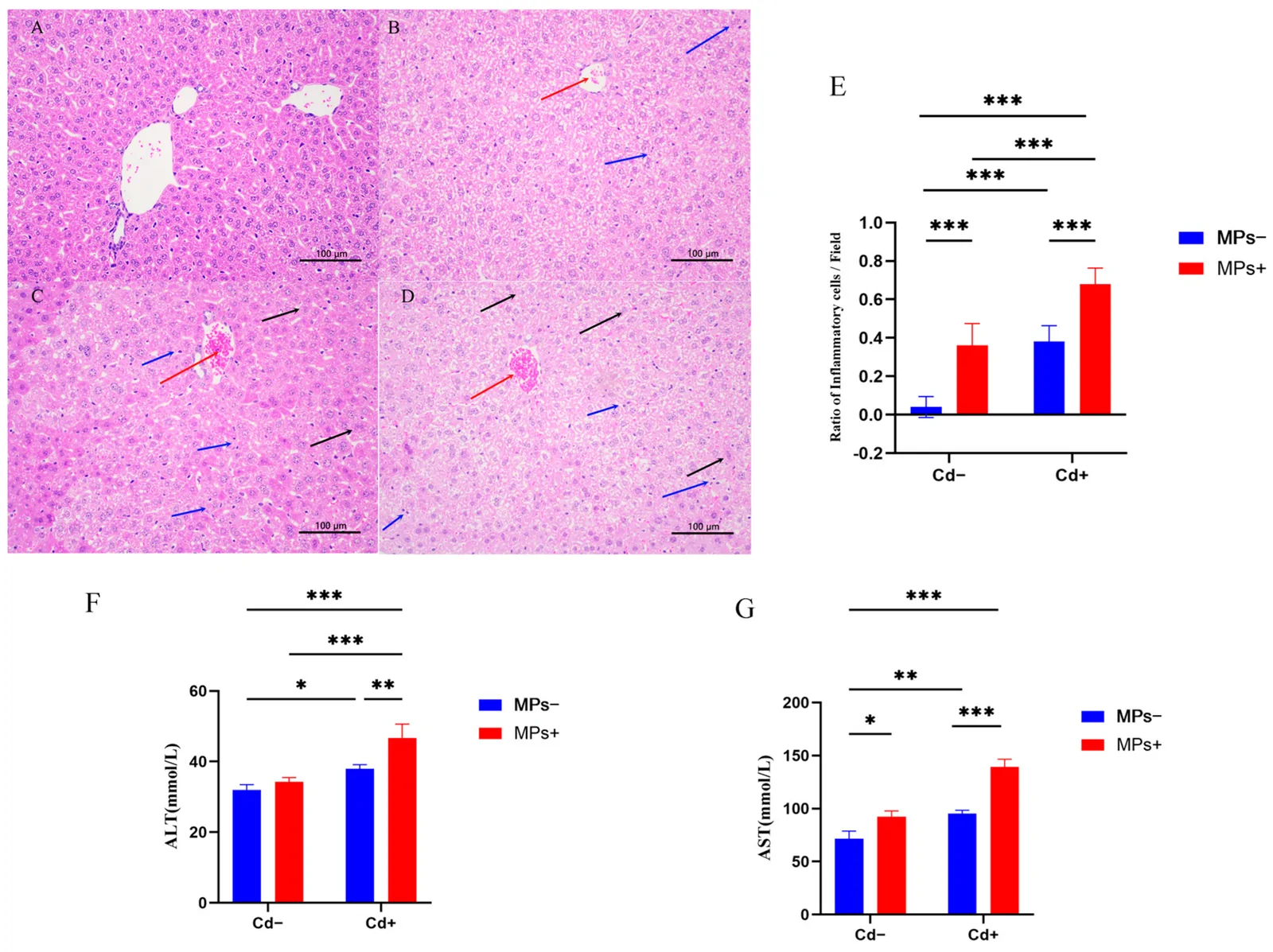
The effects of MPs on Cd-induced liver dysfunction: (**A**) Histological section of the liver from mice in the Con group; (**B**) histological section of the liver from mice in the cadmium group; (**C**) histological section of the liver from mice in the MP group; (**D**) histological section of the liver from mice in the Cd + MP group; (**E**) proportion of inflammatory cells in histological sections; (**F**) ALT levels in liver tissue; (**G**) AST levels in liver tissue. Red arrows indicate erythrocyte infiltration, blue arrows indicate inflammatory cell infiltration, and black arrows indicate hepatocyte rupture. ‘*’ indicates *p* < 0.05, ‘**’ indicates *p* < 0.01 and ‘***’ indicates *p* < 0.001.

The liver is the body’s primary metabolic organ and a key target organ for the interaction between MPs and Cd. In order to evaluate the effect of MPs on Cd-induced liver dysfunction, liver tissue was examined via H&E staining, and ALT and AST levels in the liver tissue were measured. The histopathological results indicated that, in comparison with the Con group, the Cd group exhibited substantial pathological damage to the liver tissue, primarily manifesting as the disorganization of hepatic lobule architecture, the vacuolar degeneration of hepatocytes, partial cell rupture, nuclear fragmentation, cytoplasmic vacuolization, and inflammatory cell infiltration. A small number of red blood cells were observed to be aggregating within the central veins (Figure 3A,B). The MP group also exhibited pathological changes similar to those in the Cd group (Figure 3C). In comparison to the Con group and the single-exposure groups, the Cd + MP group demonstrated the most severe liver damage, characterized by a near-complete loss of hepatic trabecular structure, indistinct hepatic sinusoids, marked hepatocyte swelling and vacuolar degeneration, exacerbated cell rupture and nuclear fragmentation, accompanied by massive inflammatory cell infiltration and red blood cell aggregation in the central veins (Figure 3A,D). A quantitative analysis of inflammatory cell infiltration was performed on H&E-stained liver tissue sections (Figure 3E). The proportion of inflammatory cells in the Cd + MP group was significantly higher than that in the Con group and the Cd group (*p* < 0.001). In comparison with the control group, both the Cd and MP groups exhibited a significant increase in the proportion of inflammatory cells (*p* < 0.05). The quantitative results indicate that combined exposure to MP and Cd induces more pronounced inflammatory cell infiltration in the liver than exposure to either substance alone. This finding is consistent with the histopathological observations described above.

ALT and AST are established biochemical markers of liver function impairment. In comparison with the Con group, both ALT and AST levels were significantly elevated in the Cd group (Figure 3F, *p* < 0.05). In the MP group, AST levels were significantly elevated (*p* < 0.05), while ALT showed only an upward trend but no significant difference. In comparison with the Con group and the Cd-exposed groups, ALT and AST levels in the Cd + MPs group were found to be significantly elevated (Figure 3F,G). The trends observed in both indicators were consistent. The results of this study indicate that MPs are capable of inducing a certain degree of liver injury and that they appear to significantly exacerbate Cd-induced liver tissue structural damage, inflammatory cell infiltration, and liver function abnormalities.

### 3.4. MPs Exacerbated Cd-Induced Oxidative Stress and Inflammatory Damage

**Figure 4:**
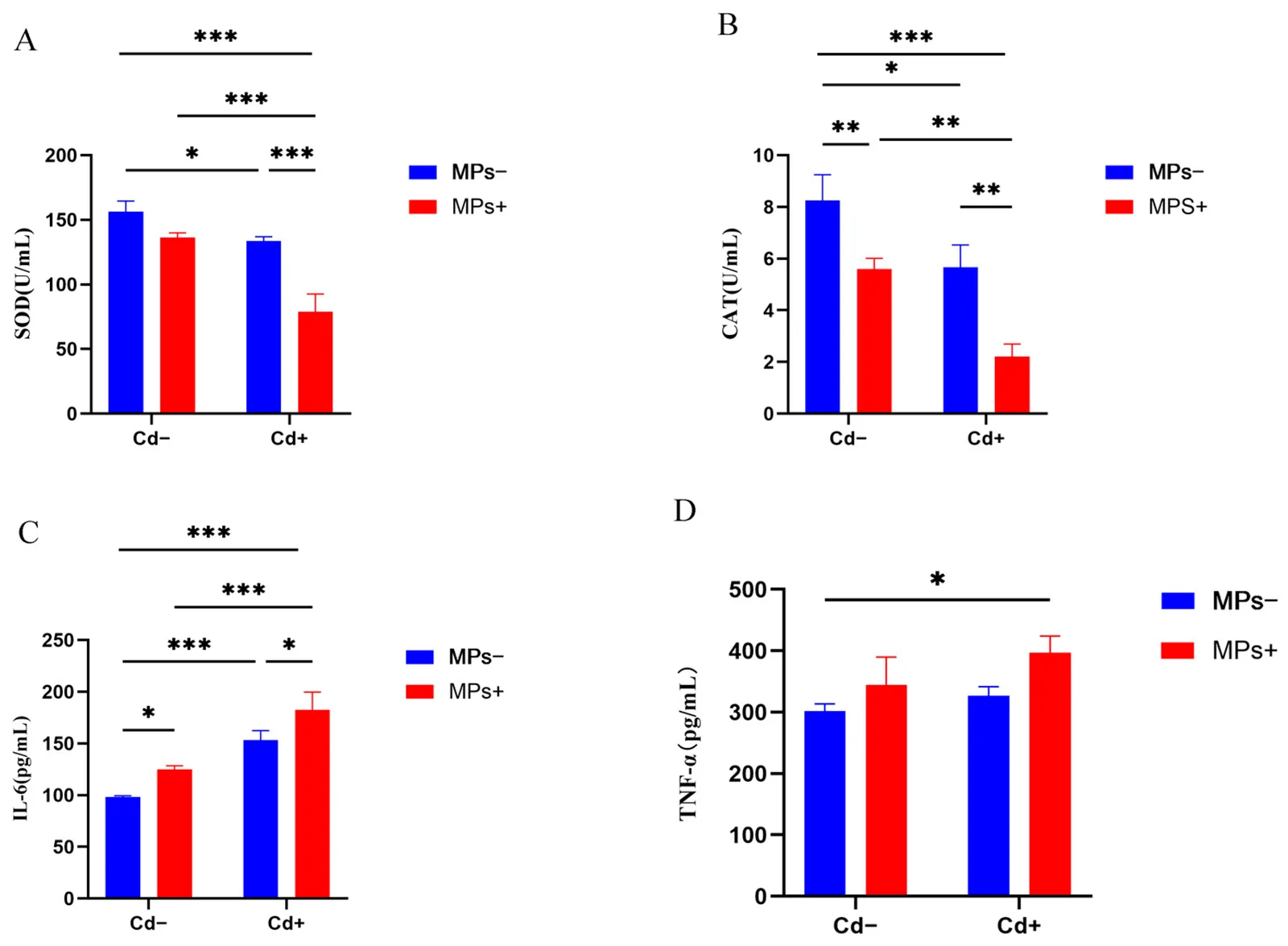
The effects of MPs on Cd-induced oxidative stress and serum inflammatory factors. (**A**) effect of MPs and Cd on SOD levels; (**B**) effect of MPs and Cd on CAT levels; (**C**) effect of MPs and Cd on TNF-α levels; (**D**) effect of MPs and Cd on IL-6 levels. ‘*’ indicates *p* < 0.05, ‘**’ indicates *p* < 0.01 and ‘***’ indicates *p* < 0.001.

Liver injury is frequently accompanied by an imbalance in oxidative stress and an enhancement of inflammatory responses. In order to ascertain the effects of MPs on Cd-induced oxidative stress and inflammatory responses, serum levels of the antioxidant markers SOD and CAT, as well as the inflammatory factors TNF-α and IL-6, were measured. The results demonstrated a significant reduction in SOD activity in both the Cd group and the Cd + MP group when compared with the Con group (Figure 4A, *p* < 0.05). While SOD activity in the MP group exhibited a downward trend, this difference did not reach statistical significance. Furthermore, CAT activity in the Cd, MP, and Cd + MP groups was significantly lower than that in the Con group (Figure 4B, *p* < 0.05). A subsequent comparison revealed that the Cd + MP group exhibited significantly lower SOD and CAT activity compared with the two single-exposure groups (*p* < 0.01). The results of the inflammatory factor assays demonstrated that the levels of IL-6 in the two single-exposure groups were significantly higher than those in the Con group (Figure 4C, *p* < 0.05). Furthermore, in comparison with the Con group, only the MP + Cd group demonstrated a significant increase in TNF-α levels; while the single-exposure group exhibited an upward trend, this was not statistically significant. (Figure 4C,D, *p* < 0.05). These results indicate that exposure to either Cd or MPs alone was associated with a certain degree of oxidative stress and inflammatory response and that the combination appeared to further amplify these effects.

### 3.5. MPs Exacerbated Cd-Induced Transcriptional Dysregulation in Mouse Livers

**Figure 5:**
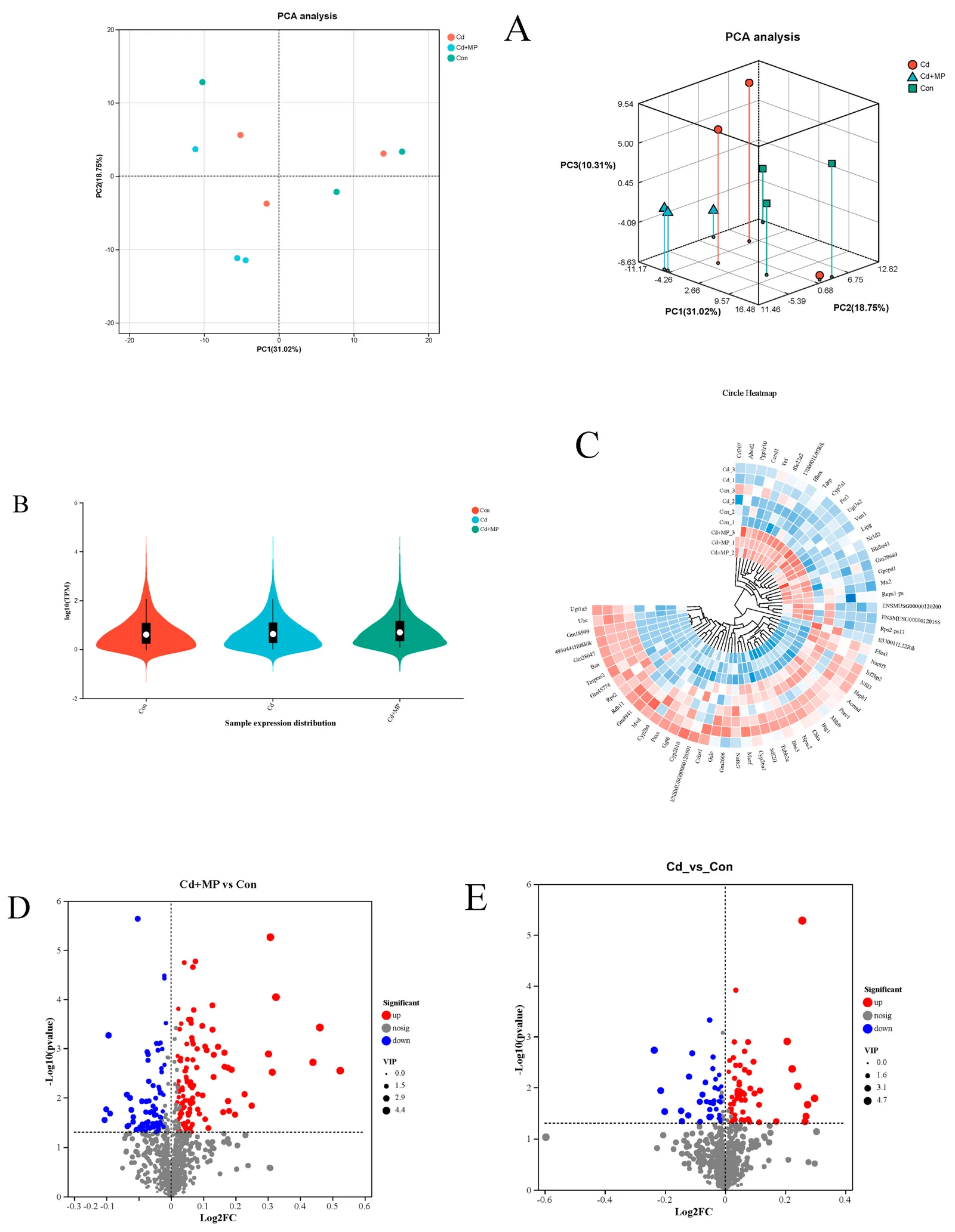

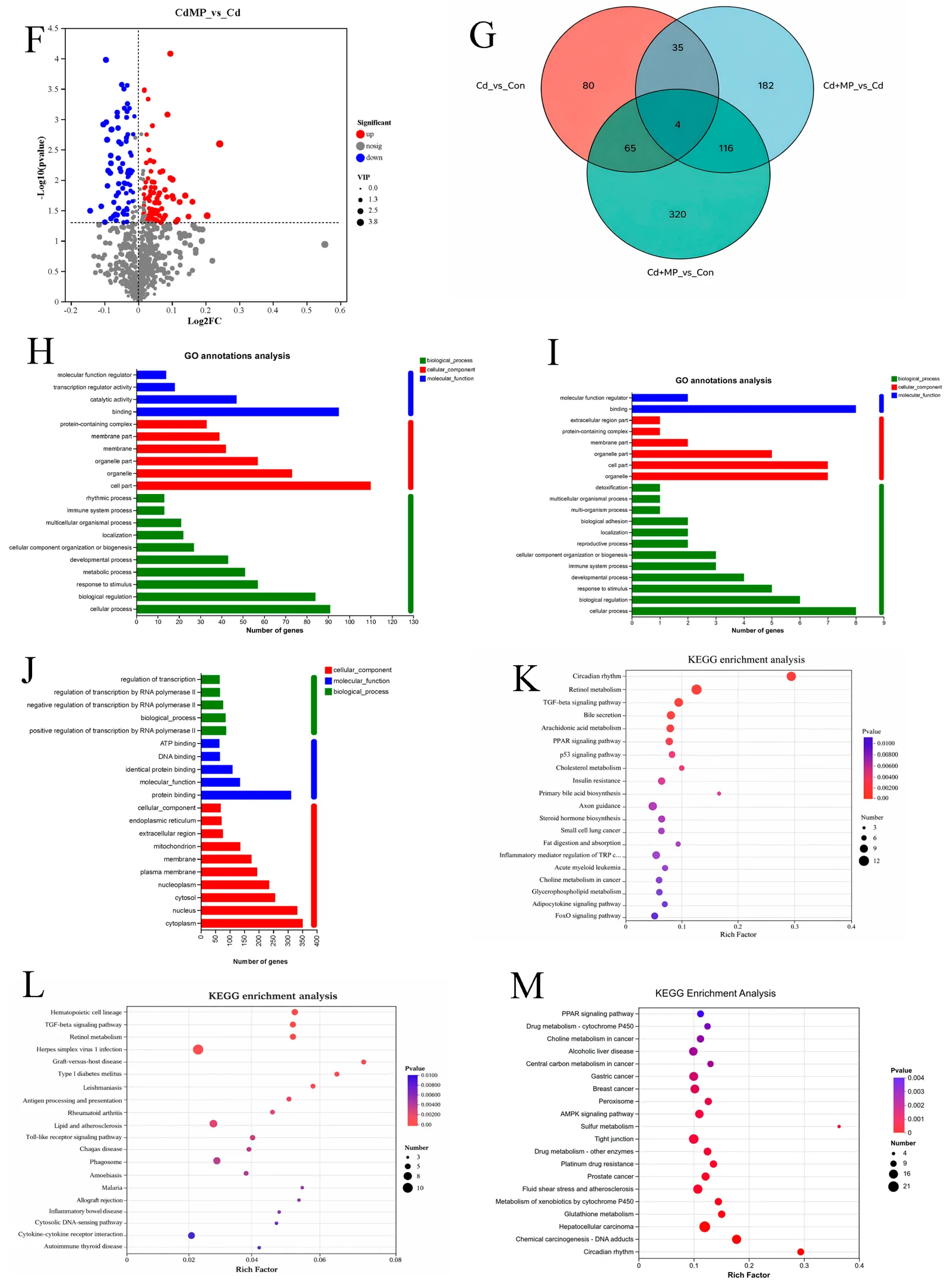
The effects of microplastics and cadmium on gene transcription in mouse livers. (**A**) Principal component analysis; (**B**) sample expression distribution; (**C**) cluster heatmap of differentially expressed genes; (**D**) volcano plot of differentially expressed genes between Cd + MP and Con groups; (**E**) volcano plot of differential genes between Cd and Con groups; (**F**) volcano plot of differentially expressed genes between Cd + MP and Cd groups; (**G**) Venn diagram; (**H**) GO analysis of differentially expressed genes between Cd + MP and Con groups; (**I**) GO analysis of differentially expressed genes between Cd and Con groups; (**J**) GO analysis of differentially expressed genes between Cd + MP and Cd groups; (**K**) the KEGG pathway enrichment results of Cd + MP vs. Con group; (**L**) the KEGG pathway enrichment results of Cd vs. Con group; (**M**) the KEGG pathway enrichment results of Cd + MP vs. Cd group.

In order to systematically elucidate the molecular mechanisms by which MPs modulate Cd-induced liver injury, transcriptomic analysis was performed to compare gene expression differences and functional enrichment patterns among the Cd + MP vs. Con, Cd + MP vs. Cd, and Cd vs. Con groups. The principal component analysis (PCA) results demonstrated that the Cd + MP group exhibited distinct characteristics compared to both the Con and Cd groups. Additionally, within the Cd + MP and Con groups, biological replicates were observed to cluster together (Figure 5A). This finding indicates the presence of adequate reproducibility in the sequencing data and suggests that the combined exposure had a significant effect on gene expression. The sample expression distribution results also demonstrated low overall dispersion among the groups (Figure 5B).

Subsequently, transcripts with a *p*-value less than 0.05 following sequencing were subjected to cluster analysis. The utilization of red and blue gradients served to denote the extent of gene upregulation and downregulation, respectively. All three groups demonstrated adequate intra-group consistency and intergroup differentiation. As illustrated by the aggregation of Cd207~Rps2-ps13 (upregulated in the Cd + MP group) into a single cluster and E530011L22Rik~Ugt1a5 (downregulated in the Cd + MP group) into another, distinctly differentiating them from the Con and Cd groups (Figure 5C). Subsequent screening for differentially expressed genes (DEGs) employing a threshold of |log2FoldChange| ≥ 1 and *p* < 0.05 revealed the identification of 505 DEGs in the Cd + MP vs. Con group, 184 DEGs in the Cd vs. Con group, and 337 DEGs in the Cd + MP vs. Cd group (Figure 5D–G). This finding suggests that co-exposure results in more extensive transcriptional disturbances compared to Cd exposure alone.

To further elucidate the biological functions of the differentially expressed genes, GO functional annotation analysis was performed on the DEGs from the Cd + MP vs. Con, Cd vs. Con, and Cd + MP vs. Cd groups. Across the three levels of biological process (BP), cellular component (CC), and molecular function (MF), the differentially expressed genes were primarily enriched in biological regulation, metabolic processes, response to stimuli/stress, and immune system-related processes. In the functional annotation analysis comparing the Cd + MP vs. Cd group, differentially expressed genes were primarily concentrated in biological processes such as energy metabolism and substance transport (Figure 5H–J). These findings suggest that, at the level of gene function, MPs may exacerbate Cd-induced liver damage by interfering with energy metabolism. Subsequent KEGG pathway enrichment analysis revealed that differentially expressed genes in the Cd + MP vs. Con group were enriched across 264 pathways, with 37 reaching significant enrichment levels. The figure displays the top 20 pathways ranked by *p*-value (Figure 5K). The pathways that were found to be significantly enriched included retinol metabolism, bile secretion, cholesterol metabolism, the AMPK signaling pathway, and the FOXO signaling pathway. A differential expression analysis of genes between the Cd and Con groups identified 163 pathways that were enriched, of which 37 exhibited significant enrichment (Figure 5L). The pathways that demonstrated the most significant enrichment primarily involved the TGF-β signaling pathway, retinol metabolism, FOXO signaling, and PPAR signaling. An investigation into the disparities in gene expression between the Cd + MP group and the Cd group was conducted, which resulted in the identification of 333 enriched pathways. Of these, 30 exhibited significant enrichment (Figure 5M). The pathways that demonstrated the most significant enrichment were primarily associated with glycerophospholipid metabolism, cholesterol metabolism, the AMPK signaling pathway, and the PPAR signaling pathway. These pathways have been demonstrated to be associated with metabolic disorders, oxidative stress, inflammatory responses, and liver dysfunction. In summary, exposure to Cd was associated with significant disruption to the hepatic transcriptional profile. Furthermore, the combination of Cd exposure and exposure to MPs appeared to expand the scope of this disruption and was associated with enhanced enrichment of relevant damage pathways.

### 3.6. MPs Exacerbated Cd-Induced Metabolic Disorders in Mouse Livers

**Figure 6:**
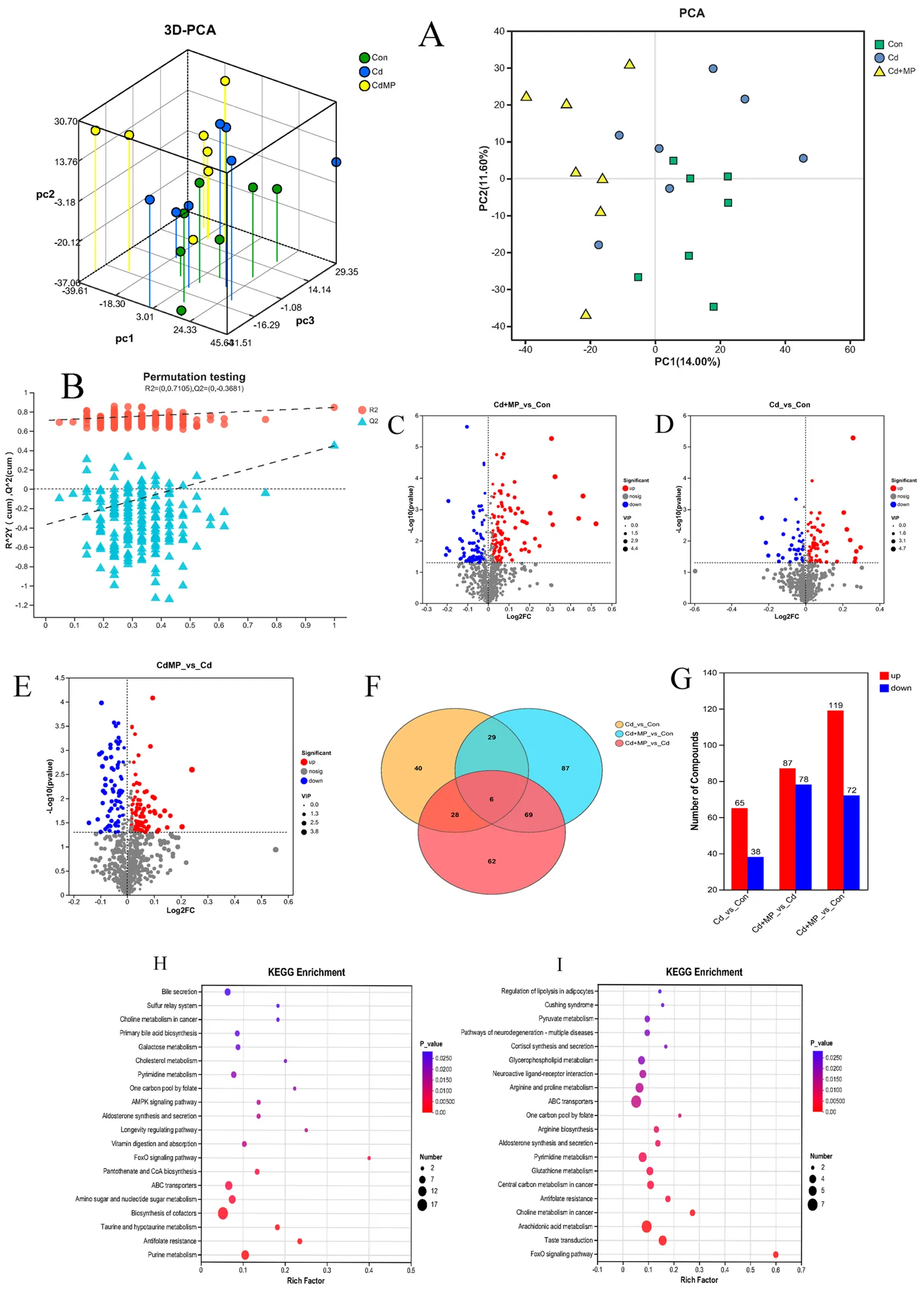
The effects of microplastics and cadmium on liver metabolites in mice. (**A**) Sample principal component analysis; (**B**) permutation testing; (**C**) volcano plot of differential metabolites between Cd + MP and Con groups; (**D**) volcano plot of differential metabolites between Cd and Con groups; (**E**) volcano plot of differential metabolites between Cd + MP and Cd groups; (**F**) differential metabolite Venn diagram; (**G**) upregulated and downregulated differential metabolite numbers; (**H**) the enrichment results of differential metabolite KEGG pathway of Cd + MP vs. Con group; (**I**) the enrichment results of differential metabolite KEGG pathway of Cd vs. Con group.

To further elucidate the metabolic effects of MPs on Cd-induced liver injury, non-targeted metabolomic analysis was performed on liver tissues from the Con group, Cd group, and Cd + MP group. PCA results indicated that samples from each group exhibited a tendency to be distinctly separated, suggesting potential differences in metabolic profiles between treatment groups. However, certain individual samples within groups demonstrated a degree of dispersion (Figure 6A). Consequently, PLS-DA was employed for discriminant analysis. The results demonstrated effective clustering of samples within each group and clear separation between groups, indicating substantial differences in metabolite profiles under varying treatment conditions. The cross-validation results indicated R2 > 0 and Q2 < 0, indicating that the model did not suffer from overfitting (Figure 6B). The identification of differentially expressed metabolites (DMs) was conducted through the implementation of a screening criterion, which included the criteria of variable importance in projection (VIP) > 1 and *p* < 0.05. The results demonstrated that a total of 103 differentially expressed metabolites were identified in the Cd vs. Con group, of which 68 were found to be upregulated and 35 were downregulated. A total of 191 differentially expressed metabolites were identified in the Cd + MP vs. Con group, including 119 that were found to be upregulated and 72 that were downregulated. Furthermore, 165 differentially expressed metabolites were identified in the Cd + MP vs. Cd group, including 87 that were found to be upregulated and 78 that were downregulated (Figure 6C–G). Among these, the Cd + MP vs. Con group exhibited the highest number of differentially expressed metabolites, suggesting that combined exposure results in a more significant disruption to hepatic metabolic homeostasis. KEGG pathway enrichment analysis revealed that differentially expressed metabolites in the Cd + MP vs. Con group were enriched across 97 metabolic pathways, with 29 reaching statistical significance. The primary pathways implicated included cholesterol metabolism, the FOXO signaling pathway, taurine and hypotaurine metabolism, primary bile acid biosynthesis, the AMPK signaling pathway, and glycerophospholipid metabolism (Figure 6H). The differential expression of metabolites in the Cd-treated group compared to the Con group was enriched across 111 pathways, with 24 of these pathways reaching statistical significance. These primarily included aldosterone synthesis and secretion, pyruvate metabolism, the FOXO signaling pathway, glutathione metabolism, ABC transporters, and glycerophospholipid metabolism (Figure 6I). The results of this study indicate that exposure to Cd was associated with changes in the composition and abundance of liver metabolites, affecting energy metabolism, lipid metabolism, cholesterol metabolism, and redox homeostasis. Furthermore, co-exposure to MPs appeared to further exacerbate the disruption of these metabolic pathways.

### 3.7. Validation of Relevant Signaling Pathways

**Figure 7:**
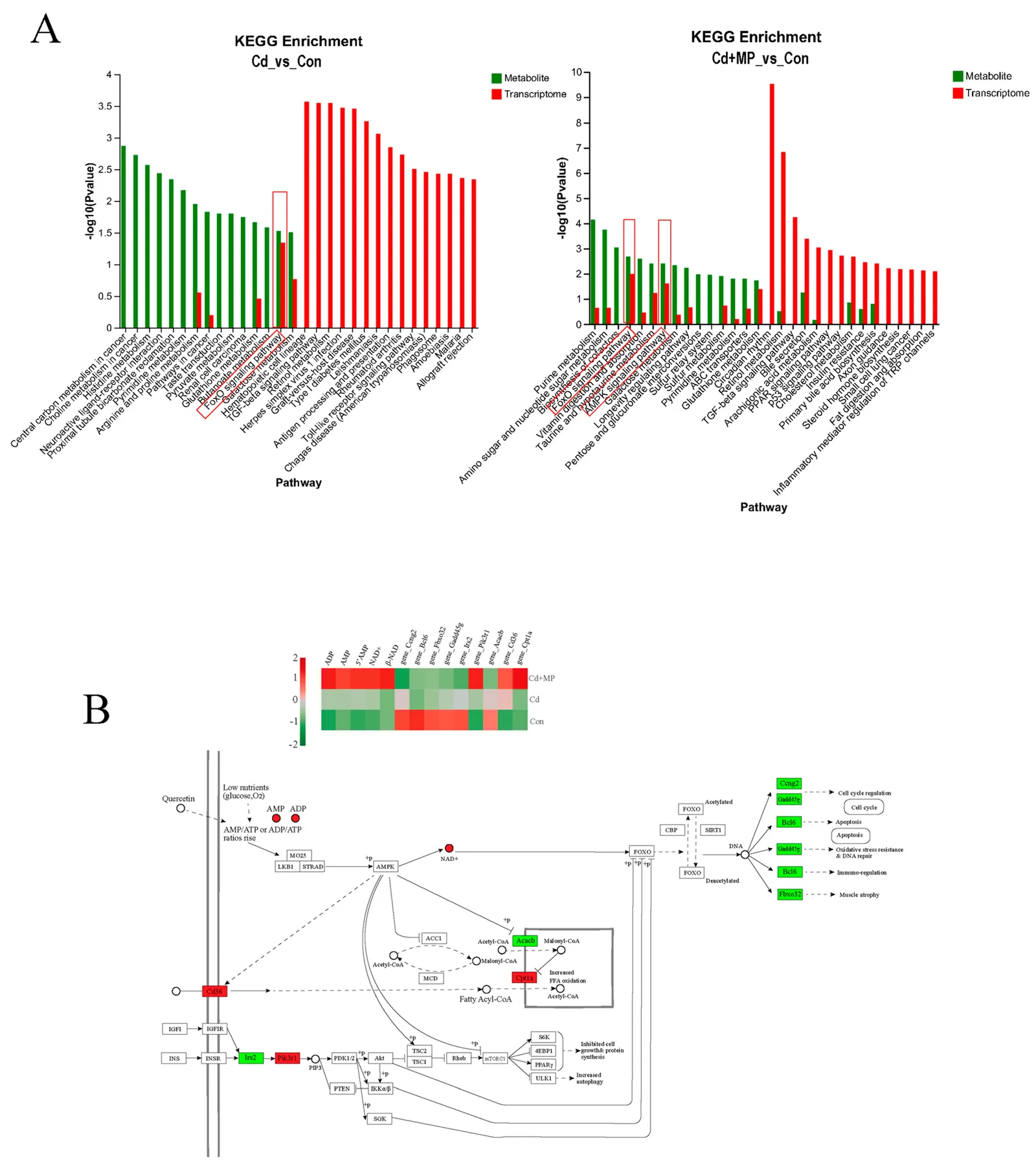

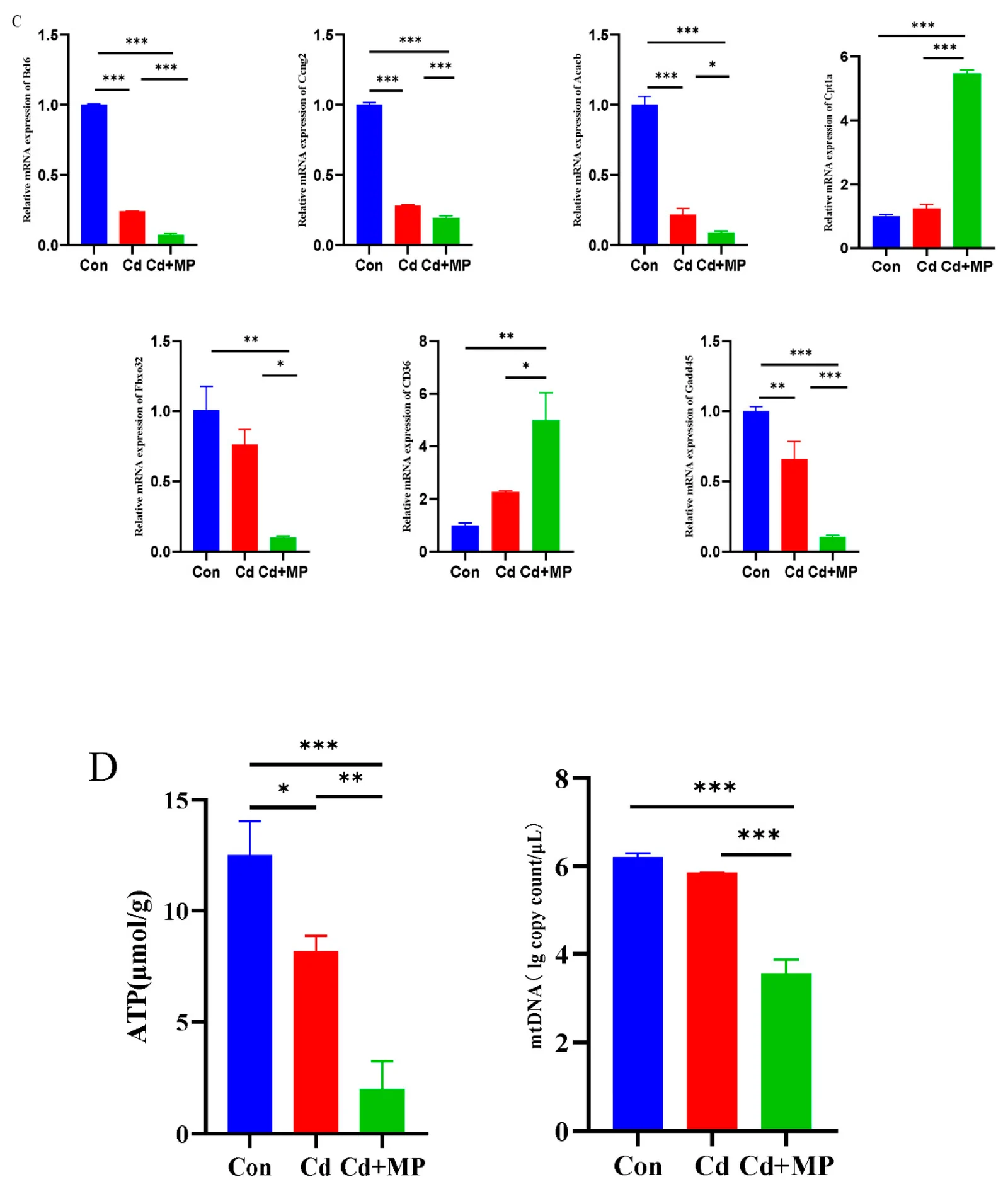
Convergence of transcriptomic and metabolomic analysis results. (**A**) The signaling pathway enriched by both transcription and metabolomics; (**B**) effects of MPs and Cd on the AMPK-FOXO signaling pathway; (**C**) validation of genes associated with the AMPK-FOXO pathway; (**D**) ATP levels and mitochondrial DNA levels. ‘*’ indicates *p* < 0.05, ‘**’ indicates *p* < 0.01 and ‘***’ indicates *p* < 0.001.

A combined analysis of transcriptomic and metabolomic enrichment results revealed that both the Cd + MP vs. Con group and the Cd vs. Con group showed significant enrichment in the FOXO signaling pathway at both the transcriptomic and metabolomic levels. This suggests that the FOXO signaling pathway may play an important role in MP- and Cd-induced liver injury in mice (Figure 7A). Furthermore, the Cd + MP vs. the Con group exhibited significant enrichment in the AMPK signaling pathway. AMPK and FOXO have been shown to be closely related in the regulation of energy metabolism in the body. Despite the fact that the Cd group did not satisfy the predefined criteria for substantial enrichment of the AMPK pathway in comparison with the Con group, the trends in changes in related genes and metabolites exhibited a high degree of consistency with those observed in the Cd + MP vs. Con group (Figure 7B). Subsequent to the acquisition of these results, qPCR was utilized to validate genes associated with the AMPK-FOXO signaling pathway. The results demonstrated that, in comparison with the Con group, Fbxo32 exhibited a significant decrease in expression only in the Cd + MP group (*p* < 0.01). Concurrently, Cd36 and Cpt1a demonstrated significant increases in expression in the Cd + MP group (*p* < 0.01). The remaining genes exhibited significant decreases in expression in both the Cd and Cd + MP groups (*p* < 0.01), with more pronounced changes observed in the Cd + MP group (Figure 7C). Subsequently, mitochondrial DNA and ATP levels were measured in order to assess the extent of impaired energy metabolism. The results demonstrated that, in comparison with the Con group, ATP levels were considerably diminished in the Cd group, while mitochondrial DNA levels exhibited only a downward tendency but no statistically significant difference. In the Cd + MP group, both mitochondrial DNA and ATP levels were significantly lower than those observed in the control and Cd groups (Figure 7D, *p* < 0.05). The findings suggest that both Cd and MPs have the potential to induce liver damage by disrupting energy metabolism-related pathways. Furthermore, the combination of these exposures appears to exacerbate AMPK-FOXO-related abnormalities and mitochondrial dysfunction, thereby leading to an aggravation of liver injury.

## 4. Discussion

Cadmium (Cd) contamination has been attributed to industrial activities such as electroplating, smelting, mining, fuel production, and battery manufacturing, which result in continuous releases of the element into the environment, thereby causing widespread contamination [26]. As demonstrated in previous studies, Cd has been found to induce liver damage through a variety of mechanisms, including apoptosis, pyroptosis, inflammatory responses, and interference with metabolic pathways [27,28]. The presence of MPs in soil and water bodies is a consequence of various processes, including photodegradation of plastics, physical abrasion, and their incorporation as additives in personal care products. These MPs pose a potential threat to animal and human health through bioaccumulation in the food chain. Due to their substantial specific surface area and robust adsorption capacity, MPs have the capacity to carry toxic and harmful substances and heavy metals from the environment, thereby enhancing their biological toxicity. The majority of extant studies on the combined toxicity of MPs and Cd have focused on aquatic organisms, including fish, algae and aquatic mollusks [29]. Research on mammalian toxicity, particularly liver toxicity, remains relatively limited, and the underlying molecular mechanisms have not yet been fully elucidated. Therefore, this study established a mouse model by gavaging mice with PS-MPs and CdCl_2_ and systematically investigated the toxicological mechanisms through transcriptomic and metabolomic analyses. The findings suggest that exposure to MPs and Cd, either individually or in combination, may contribute to liver damage. However, exposure to the combination leads to a more significant increase in liver injury. Further multi-omics analysis suggests that energy metabolism disorders, mediated by the AMPK-FOXO signaling axis, may represent an important mechanism through which MPs could exacerbate Cd-induced liver damage, although this inference requires further validation.

The present study provided further evidence for the hepatotoxic effects of MPs and Cd at the phenotypic and histopathological levels. We observed PS-MPs using a scanning electron microscope and found them to be spherical with a smooth surface and regular arrangement. The adsorption of Cd onto MPs has been demonstrated to result in alterations to the material’s surface properties, manifesting in the form of roughness, cracking, and an uneven particle size distribution. These changes in surface characteristics have been shown to enhance the materials’ adsorption capacity and their propensity for biological interactions [30,31]. The combination of MPs and Cd, either in isolation or in conjunction, has been demonstrated to elicit an elevated level of ROS, which in turn can inflict damage to cell membranes, proteins, and DNA molecules. The augmented energy expenditure that is necessitated to restore tissue and cellular homeostasis has been shown to result in a decline in growth performance in animals [32]. In this study, the weight gain of mice in all single-agent poisoning groups was significantly reduced in comparison with the blank group. The findings revealed no substantial disparities among the single-agent poisoning groups. The weight gain of mice in the combined poisoning group was significantly lower than that of the other three groups, which is consistent with the results of Hu et al.’s study on combined poisoning in mice [33]. H&E staining revealed that the liver lobules and hepatocytes in the single Cd or MP exposure groups exhibited some degree of pathological changes; however, in the combined exposure group, the liver lobules and sinusoids were completely obscured, hepatocytes were extensively ruptured, and there was massive infiltration of inflammatory cells. These findings are consistent with the hypothesis that MPs exacerbated Cd-induced liver damage in mice. In light of the findings from our previous study, which demonstrated that combined exposure results in intestinal barrier damage in mice [34], as well as the reports of enhanced fluorescent MP signals in the liver of a combined exposure model in ducks [30], it is plausible that MPs may, in one respect, facilitate the transport and accumulation of Cd within the body through a hypothesized ‘carrier effect,’ though this proposed mechanism remains to be directly validated through Cd quantification in target tissues. Additionally, given the observed alterations in the physicochemical properties of MPs following co-treatment with Cd, there is a possibility that this may enhance the “carrier effect.” Furthermore, in natural environments, microplastics are subject to various external factors, including light, heat, physical forces, and friction, which can lead to the development of a phenotype characterized by variable sizes and rough surfaces. In this state, microplastics may have an augmented capacity to carry heavy metals, thereby promoting the transport and accumulation of Cd within the body. In another respect, MPs may increase the probability of Cd entering the portal venous circulation and reaching the liver due to intestinal barrier damage. In accordance with these findings, the heightened levels of ALT and AST, accompanied by the diminished levels of SOD and CAT, and the augmented levels of TNF-α and IL-6, as observed in this investigation, suggest that concomitant exposure engenders more substantial liver dysfunction, an imbalance in oxidative stress, and heightened systemic inflammatory responses. These findings are generally consistent with the conclusions of Sun et al.’s findings regarding duck pancreas [35]. It must be acknowledged that ALT and AST are measured in liver tissue homogenates, rather than in serum, with the aim of directly assessing the enzyme activity retained within the liver tissue. This approach reflects the functional metabolic capacity of hepatocytes following exposure to toxins. However, it is imperative to exercise caution when interpreting these results, as the impact of the homogenization process on these markers cannot be overlooked. The collective findings concerning body weight gain, liver index, histological changes, and biochemical indicators suggest that both MPs and Cd are hepatotoxic, with combined exposure associated with a more pronounced damaging effect, consistent with a potential synergistic interaction.

To further elucidate the molecular basis of Cd-induced liver injury and the exacerbation of its toxicity by MPs, we performed transcriptomic analysis on liver tissues from mice in the Con, Cd, and MP + Cd groups. The results demonstrated significant disparities in the transcriptional expression among the three distinct groups. In comparison with Cd exposure alone, combined exposure resulted in a greater number of differentially expressed genes and a broader range of enriched pathways, primarily involving processes such as inflammatory responses, oxidative stress, lipid metabolism, and energy metabolism. Specifically, the TGF-β signaling pathway, retinol metabolism, the PPAR signaling pathway, and the FOXO signaling pathway exhibited increased enrichment in the combined exposure group, suggesting that MPs may not simply add to the toxicity of Cd but could further amplify the disruption of molecular networks closely associated with liver injury.

The TGF-β signaling pathway plays a pivotal regulatory role in the progression of inflammation and the development of fibrosis following liver injury. Damage to the liver results in the secretion of transforming growth factor-β1 (TGF-β1) by non-parenchymal cells, such as Kupffer cells. This leads to the activation of hepatic stellate cells through Smad-dependent or non-Smad pathways, which in turn increases the levels of α-smooth muscle actin (α-SMA), collagen, and other extracellular matrix components, thereby promoting the fibrotic process [36]. As demonstrated in prior research, an excess of ROS has been observed to induce the activation of the TGF-β latent complex by modifying its conformation [37]. In this study, TNF-α levels exhibited a significant increase following Cd exposure, while transcriptomic results indicated substantial enrichment of the TGF-β pathway. These findings suggest that Cd-induced oxidative stress and the inflammatory microenvironment may promote the activation of this pathway. According to the findings of other studies, the presence of Cd has been demonstrated to exert an influence on the expression of TGF-β-related molecules through downregulation of microRNAs (miRNAs), including but not limited to miR-26a and miR-21. This, in turn, results in alterations in cell migration and stress responses [38]. In a chicken liver cell model exposed to Cd, Cd significantly activated the TGF-β pathway, leading to tissue fibrosis. These observations are consistent with the results of our study. In light of these findings, we hypothesize that Cd may exacerbate TGF-β-related damage processes through several mechanisms, including ROS accumulation, inflammatory cytokine release, and post-transcriptional regulation. Furthermore, studies have demonstrated that concomitant exposure to MPs may amplify the activation of this pathway via the mechanisms outlined above.

Abnormal retinol metabolism may also be a key mechanism underlying Cd- and MP-induced liver injury. Vitamin A and its metabolite, retinoic acid (RA), play a crucial role in maintaining hepatocyte differentiation, lipid metabolism homeostasis, and the immune-inflammatory balance. Metabolic imbalances in these processes are closely associated with hepatocyte injury, inflammation, and fibrosis [39]. Cyp26 is a key enzyme in the metabolism and clearance of RA, a process that can reduce intracellular RA levels [40]. The transcriptomic results of this study indicate that cadmium exposure upregulates Cyp26-related expression, suggesting that RA clearance may be enhanced, thereby weakening its role in maintaining inflammation and metabolic homeostasis. As indicated by the findings of previous studies, there is a possibility that abnormalities in retinol/retinoic acid metabolism interact with inflammatory signaling pathways such as NF-κB [41]. Furthermore, extant research has demonstrated that aberrant expression of the CYP26 family is intimately associated with pathological processes involving liver inflammation [42]. In an alcoholic fatty liver disease model, decreased levels of RA in the liver were accompanied by increased expression of the Cyp26a1 and Cyp26b1 genes, which is consistent with the trends observed in this study [43]. Consequently, exposure to Cd, in conjunction with MPs, has the potential to exacerbate hepatic inflammation and injury by disrupting retinol metabolism.

The abnormal activation of the peroxisome proliferator-activated receptor (PPAR) signaling pathway suggests the involvement of lipid metabolic reprogramming in the liver injury process observed in this study. The PPAR family comprises a set of critical nuclear receptor transcription factors that detect fatty acids and their derivatives, thereby regulating lipid metabolism, inflammatory responses, and energy homeostasis [44]. In this study, the analysis of pathway-related genes revealed an increased expression of Cyp4a14 and Cd36 under conditions of cadmium exposure, both in isolation and in combination with other stressors. As a PPARα target gene, the Cyp4a family is involved in the ω-hydroxylation of fatty acids. Its excessive activation may be accompanied by increased ROS production and exacerbate oxidative damage [45]. Concurrently, CD36, functioning as a fatty acid transport receptor, has been demonstrated to promote the uptake of free fatty acids by hepatocytes and induce lipid deposition. Moreover, it has been shown to drive the progression of fatty liver disease through lipid synthesis pathways, such as SREBP1 [46]. Furthermore, macrophages exhibit an increased propensity to transition toward a pro-inflammatory phenotype following the internalization of oxidized lipids via CD36. Additionally, exposure to nano- and microplastics has been demonstrated to induce morphological and lipid metabolic abnormalities in macrophages, thereby promoting an inflammatory shift [47]. Therefore, combined exposure to MPs and Cd may promote lipid accumulation, oxidative damage, and inflammatory amplification through the PPAR-CD36-related network, thereby exacerbating liver damage.

The results of the metabolomics study further corroborated the aforementioned inferences. In comparison with the Con group, 191 and 103 differentially expressed metabolites were identified in the Cd + MP group and the Cd group, respectively. Both groups exhibited significant enrichment in pathways associated with oxidative stress and energy metabolism, including aldosterone synthesis and secretion, ABC transporters, glycerophospholipid metabolism, pyrimidine metabolism, and the FOXO signaling pathway. Glycerophospholipid metabolism plays a pivotal role in preserving membrane integrity and maintaining lipid homeostasis. Disruptions in this process can lead to the accumulation of lipid droplets, inflammatory activation, and subsequent tissue damage [48]. In this study, 3-phosphoglycerate (3-PGA) exhibited a significant increase, indicating that under toxic stress conditions, the allocation of carbon flux between glycolytic intermediates and membrane lipid synthesis may have undergone reorganization. ABC transporters are widely expressed in the liver and facilitate the transport of various endogenous and exogenous substrates via ATP [49]. Alterations in these pathways, in conjunction with elevated levels of metabolites such as taurine and glutamate, suggest that the liver initiates detoxification and antioxidant compensatory responses following toxin exposure. Concurrently, elevated levels of pyrimidine metabolism-related molecules, such as thymidine, uridine-5′-monophosphate (UMP), and UDP-glucose, suggest that liver tissue exhibits significant stress adaptation in terms of DNA damage repair, nucleotide resynthesis, and energy substrate redistribution [50]. In summary, simultaneous exposure to MPs and Cd has been shown to induce more widespread and pronounced metabolic imbalances.

It is noteworthy that in the pathways involved in aldosterone synthesis and secretion, there was an enrichment of differentially expressed metabolites such as AMP and NAD+ in the AMPK-FOXO-related pathways. Similarly, in the transcriptomic analysis, the Cd + MP vs. Con group exhibited significant enrichment in the AMPK and FOXO signaling pathways. While the Cd vs. Con group was not statistically significantly enriched in the AMPK pathway, the direction of changes in its associated genes and metabolites was largely consistent with that of the co-exposed group. The AMPK-FOXO axis has been demonstrated to be intimately linked with energy balance and stress resistance [51]. Research has demonstrated that cadmium disrupts glucose and lipid metabolism in the kidneys, induces oxidative stress, and triggers inflammation. However, luteolin has been shown to exert a protective effect by activating the AMPK/SIRT1/FOXO1 pathway [52]. Furthermore, a reduction in AMPK phosphorylation levels was observed in the livers of mice administered PS-MPs orally, and a significant decrease in FOXO1 expression was detected in the ovaries of zebrafish exposed to PS-MPs [53,54]. This suggests that energy stress may be an important basis for Cd-induced hepatotoxicity, with MPs further exacerbating this change. Consequently, this study further validated AMPK-FOXO-related energy metabolism abnormalities using qPCR, mitochondrial DNA, and ATP content assays.

AMPK is a pivotal molecule implicated in cellular energy sensing; it is activated when the AMP/ATP ratio increases and further regulates the FOXO family of transcription factors, thereby participating in processes such as the oxidative stress response, DNA repair, and cell cycle regulation [55]. In this study, the genes associated with the AMPK-FOXO pathway—Bcl6, Gadd45, Ccng2, Acacb, Cpt1a, Fbxo32, and Cd36—exhibited varying degrees of abnormal expression. Bcl6 has been shown to inhibit pro-inflammatory transcription, and its downregulation is often associated with a diminished capacity to suppress inflammation [56]. Cd may suppress Bcl6 expression by activating the NF-κB inflammatory signaling pathway, while MPs may indirectly inhibit Bcl6 expression by disrupting endoplasmic reticulum function [57]. Growth arrest and DNA damage-inducible 45 (Gadd45) and cyclin G2 (Ccng2) have been identified as critical factors in the DNA damage response and cell cycle negative regulation, respectively. Their downregulation in the context of persistent toxic stress suggests that the repair and regulatory capabilities of hepatocytes may be compromised [43,58]. The qPCR trends for bcl-6 and Ccng2 were contrary to those observed in the transcriptome. This phenomenon may be attributed to post-transcriptional regulatory mechanisms, differences in detection sensitivity between the two platforms (RNA-seq quantification versus qPCR amplification efficiency), and the fact that these two genes are involved in anti-inflammatory and cell cycle regulation, respectively. Compared with other members of the pathway, their expression may be influenced by more complex regulatory factors, leading to the observed inter-platform variability. It is noteworthy that for all genes that have been validated, the overall trend of downregulation remained consistent across both platforms. This finding lends further support to the hypothesis that the AMPK-FOXO pathway is suppressed under combined exposure conditions. In addition, the downregulation of Acacb and the upregulation of Cpt1a represent a classic inverse relationship, indicating that under energy-deprived conditions, hepatocytes tend to suppress fatty acid synthesis and enhance fatty acid β-oxidation to maintain energy supply [59]. Concurrently, the downregulation of Fbxo32 suggests impaired protein degradation and stress adaptation, while the upregulation of Cd36 further indicates increased lipid uptake and amplified inflammation [60,61]. These findings demonstrate that combined exposure to Cd and MPs promotes metabolic imbalance and the progression of liver cell damage by reshaping the AMPK-FOXO-related gene network.

Moreover, the decline in mitochondrial DNA content and ATP levels is consistent with impaired energy metabolism. Mitochondria play a pivotal role in maintaining energy homeostasis in hepatocytes. Sustained involvement of the AMPK-FOXO axis is frequently associated with impaired mitochondrial function and elevated metabolic stress [62,63]. In comparison with the Cd group, the Cd + MP group exhibited a significant decrease in mitochondrial DNA and ATP levels in liver tissue, suggesting that combined exposure results in more pronounced mitochondrial impairment and energy deficiency. A comprehensive review of the extant literature, in conjunction with pathological observations, multi-omics analysis, and qPCR validation results, suggests that one of the primary mechanisms by which microplastics exacerbate cadmium-induced liver damage involves the further aggravation of energy metabolism disorders and the induction of mitochondrial dysfunction. It is important to note that the present study primarily elucidated the involvement of the AMPK-FOXO axis at the transcriptional and metabolic levels. Further validation of the causal relationship is required through protein-level, phosphorylation-status, and cell-specific experiments.

Several limitations of the present study should be acknowledged when interpreting the findings. First, and most importantly, we did not directly measure the concentrations of cadmium or microplastics in the liver tissue of treated mice. Consequently, the link between the doses, hepatic accumulation of these toxicants, and the observed pathological and molecular changes cannot be established with certainty. The conclusions drawn in this study regarding the “carrier effect” of MPs on Cd and the exacerbation of Cd-induced hepatotoxicity by MPs are therefore based on indirect inferences from phenotypic, biochemical, transcriptomic, and metabolomic data, supported by published evidence demonstrating hepatic Cd deposition under comparable exposure regimens. Future studies should incorporate quantitative analytical methods, such as inductively coupled plasma mass spectrometry (ICP-MS) for Cd measurement and pyrolysis-gas chromatography/mass spectrometry (Py-GC/MS) or fluorescent labeling for MP quantification, to establish direct exposure–accumulation–damage relationships. Second, while scanning electron microscopy was performed on pristine PS-MPs, we did not characterize the surface properties of Cd-adsorbed PS-MPs. Given that different polymer types (e.g., polystyrene vs. polyvinyl chloride) exhibit distinct surface chemistries and adsorption affinities for heavy metals, the extrapolation of findings from studies using other polymer types should be made with caution. Third, the multi-omics analyses in this study were conducted at the transcriptional and metabolic levels; protein-level validation of the AMPK-FOXO signaling axis, including phosphorylation status, was not performed. The causal role of this pathway in MP + Cd-induced hepatotoxicity requires further confirmation through targeted protein analyses and pathway inhibition/activation experiments. Despite these limitations, we believe the present study provides valuable mechanistic insights into the hepatic effects of combined MP and Cd exposure in a mammalian model.

In summary, taken together, our data suggest that the presence of MPs may significantly amplify the toxic effects of Cd on the mouse liver. The underlying mechanism may involve not only the exacerbation of oxidative stress and inflammatory responses but also multifaceted disruptions in retinol metabolism, PPAR-related lipid metabolism, and AMPK-FOXO-mediated energy metabolism reprogramming. However, given that hepatic Cd concentrations were not directly measured in this study, these mechanistic inferences should be interpreted with caution and require further experimental validation.

## 5. Conclusions

The present study provided evidence that exposure to either MPs or Cd, either individually or in combination, was associated with structural and functional damage to the mouse liver, triggering oxidative stress and eliciting inflammatory responses. However, the damage observed under combined exposure was more severe. Integrative transcriptomic and metabolomic analyses identified abnormalities in retinol metabolism, PPAR/FOXO-related lipid metabolism, and energy metabolism—particularly those centered on the AMPK-FOXO signaling axis—as prominent molecular features associated with Cd + MP-induced liver injury. The integration of qPCR, mitochondrial DNA, and ATP analysis provides additional support for the hypothesis that MPs may potentiate Cd-induced hepatic toxicity in murine models, in part by exacerbating energy metabolism imbalance. Nevertheless, further studies incorporating direct quantification of hepatic Cd and MP levels, protein-level validation, and functional perturbation experiments are warranted to establish the causal relationships suggested by these findings.

## Figures and Tables

**Table 1:** Experimental groups and treatments.

Group	Gastric Lavage Solution
Control group	Distilled water
Cd group	4.8 mg/kg/d CdCl_2_
MP group	10 mg/kg/d MPs
Cd + MP group	4.8 mg/kg/d CdCl_2_ + 10 mg/kg/d MPs

**Table 2:** Primer sequences used for qRT-PCR analysis.

Gene	Direction (5′-3′)	Sequence
Cpt1a	FORWARD	ACAACAACGGCAGAGCAGAGG
	REVERSE	GGACACCACATAGAGGCAGAAGAG
Cd36	FORWARD	TTGCGACATGATTAATGGCACAGAC
	REVERSE	AGATCCGAACACAGCGTAGATAGAC
Acacb	FORWARD	GATCGCCTCCACCATCGTAGC
	REVERSE	TGTCCTCCGTCCACTCCACTG
Pik3r1	FORWARD	GGGAGCAGCAACCGAAACAAAG
	REVERSE	CCACTACGGAGCAGGCATAGC
Irs2	FORWARD	TGGATAGAGGACTGAGGAAGAGGAC
	REVERSE	GGCGACCTGAACTACCAGAGAAG
Gadd45g	FORWARD	GTCTACGAGTCCGCCAAAGTCC
	REVERSE	CACAGCAGAACGCCTGAATCAAC
Fbxo32	FORWARD	GCCTCACGATCACCGACCTG
	REVERSE	GCAGAGTCTCTTCCACAGTAGCC
Bcl6	FORWARD	TCGTGAGGTCGTGGAGAACAATATG
	REVERSE	GAAGGTGCTGAGCGGGAGATG
Ccng2	FORWARD	CAGAACCTCCACAGCAGCTACTAC
	REVERSE	TCCTCTCCACAACTCATGTCTTCAC

## Data Availability

The original RNA-seq data presented in this study are openly available in in the NCBI BioProject/SRA database at https://www.ncbi.nlm.nih.gov/sra/PRJNA1476926 (accessed on 20 June 2026), reference number PRJNA1476926. The original untargeted metabolomics data have been deposited in the MetaboLights database and will be openly available under accession number [MTBLS20260610220504] once validation is completed. Other data supporting the findings of this study are included in the article. Further inquiries can be directed to the corresponding author.
